# Morphometry of two cryptic tree frog species at their hybrid zone reveals neither intermediate nor transgressive morphotypes

**DOI:** 10.1002/ece3.8527

**Published:** 2022-01-27

**Authors:** Tomasz Majtyka, Bartosz Borczyk, Maria Ogielska, Matthias Stöck

**Affiliations:** ^1^ Department of Evolutionary Biology and Conservation of Vertebrates University of Wrocław Wrocław Poland; ^2^ Leibniz‐Institute of Freshwater Ecology and Inland Fisheries (IGB) Berlin Germany; ^3^ Amphibian Research Center Hiroshima University Higashi‐Hiroshima Japan

**Keywords:** amphibians, hybridization, hybrid zones, *Hyla arborea* group, Hylidae, morphometics, transgressive phenotypes, transgressive segregation

## Abstract

Under incomplete reproductive isolation, secondary contact of diverged allopatric lineages may lead to the formation of hybrid zones that allow to study recombinants over several generations as excellent systems of genomic interactions resulting from the evolutionary forces acting on certain genes and phenotypes. Hybrid phenotypes may either exhibit intermediacy or, alternatively, transgressive traits, which exceed the extremes of their parents due to epistasis and segregation of complementary alleles. While transgressive morphotypes have been examined in fish, reptiles, birds, and mammals, studies in amphibians are rare. Here, we associate microsatellite‐based genotypes with morphometrics‐based morphotypes of two tree frog species of the *Hyla arborea* group, sampled across a hybrid zone in Poland, to understand whether the genetically differentiated parental species also differ in morphology between each other and their hybrids and whether secondary contact leads to the evolution of intermediate or transgressive morphotypes. Using univariate approaches, explorative multivariate methods (principal component analyses) as well as techniques with prior grouping (discriminant function analyses), we find that morphotypes of both parental species and hybrids differ from each other. Importantly, hybrid morphotypes are neither intermediate nor transgressive but found to be more similar to *H*. *orientalis* than to *H*. *arborea*.

## Graphical abstract

We associate microsatellite‐based genotypes with morphometrics‐based morphotypes of two tree frog species of the *Hyla arborea* group, sampled across a hybrid zone in Poland, to understand whether the genetically differentiated parental species also differ in morphology between each other and their hybrids and whether secondary contact leads to the evolution of intermediate or transgressive morphotypes. We find that morphotypes of both parental species and hybrids differ from each other. Importantly, hybrid morphotypes are neither intermediate nor transgressive but found to be more similar to *H. orientalis* than to *H*. *arborea*.

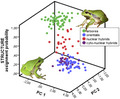

## 1 INTRODUCTION

Where allopatric evolutionary lineages, including cryptic species, come into secondary contact, depending on their genetic distance and other complex intrinsic and extrinsic factors, the most extreme outcomes may either be “complete” reproductive isolation with no or sterile offspring, or “merging” of both gene pools (Rudman & Schluter, 2016 incl. refs.). At intermediate evolutionary stages, however, both lineages may rather form hybrid zones (Harrison, 1990; Maroja et al., 2015; Payseur & Rieseberg, 2016). In the latter ones, various recombinants over several generations present excellent systems to study natural organismal genomic interactions as a result of the evolutionary forces acting on certain genes and phenotypes (Abbott et al., 2013) and reflecting the dispersal of the animals (Barton & Hewitt, 1985). Hybrids may often exhibit intermediate phenotypes, compared to their parental lineages (Kierzkowski et al., 2011, 2013; Szymura, 1993), including poor adaptations to their ancestral ecological niches (Svedin et al., 2008; Vamosi et al., 2000). Alternatively, hybrid phenotypes, from microbes to vertebrates, can exhibit so‐called transgressive traits, which exceed the extremes in their parents along a huge character spectrum, for example, affecting morphology, behavior, or ecological niches. Transgression is explained by epistasis and segregation of complementary alleles (transgressive segregation; Rieseberg et al., 1999; Slatkin & Lande, 1994; Stelkens et al., 2009 and refs. therein; Abbott et al., 2013; Ficetola & Stöck, 2016; Pereira et al., 2014) and might promote adaptive radiations (Kagawa & Takimoto, 2018).

In vertebrates, transgressive morphotypes have been examined in fish (Stelkens et al., 2009), reptiles (Robbins et al., 2014), birds (Campagna et al., 2018), and mammals (Boel et al., 2019; Larsen et al., 2010). Morphometric studies in amphibian hybrid zones have long been focusing on the variation in transects or rarely associating morphotypes and genotypes, but then, to our knowledge, mostly without investigating the occurrence of transgressive morphotypes (Babik & Rafiński, 2004; Fijarczyk et al., 2011; Gollmann et al., 1988; Kuchta, 2007). Here, we study an anuran hybrid zone by associating microsatellite‐based genotypes and morphometrics‐based morphotypes in tree frogs of the *Hyla arborea* group (*sensu* Faivovich et al., 2005) to understand whether secondary contact leads to the evolution of transgressive morphotypes.

Our focal system is a hybrid zone of tree frogs (*Hyla arborea*/*H*. *orientalis*) in eastern Central Europe. *Hyla arborea* (Linnaeus, 1758) has long been considered the only tree frog species inhabiting Western, Central and Eastern Europe. Phylogenetics, involving mitochondrial and nuclear DNA, revealed *H*. *orientalis*, its Iberian sister species *H*. *molleri* and three initially unnamed new cryptic taxa (Dufresnes et al., 2018, 2019; Gvoždík et al., 2010; Stöck et al., 2008). Stöck et al. (2012) suggested at least a Pliocene origin of *H*. *orientalis* and *H*. *arborea*, that both exhibit molecular signatures of a postglacial range expansions and found that *Hyla arborea* meets different mtDNA‐clades of *H*. *orientalis* “in NE‐Greece, along the Carpathians, and in Poland along the Vistula River (there including hybridization).” Dufresnes et al. (2013) further characterized the population structure of *H*. *arborea* with only one genetically depauperate lineage that postglacially recolonized Central and Western Europe. Likewise, of the genetically much more divers *H*. *orientalis*, only a single mtDNA‐lineage postglacially recolonized northeastern Europe (Dufresnes, Litvinchuk, et al., 2016; Stöck et al., 2012). Thus, only two single mitochondrial haplotype groups of both none‐sister taxa, *H*. *arborea* and *H*. *orientalis* in parapatric distribution, form the ca. 100 km wide hybrid zone in the lowlands along the Vistula River of Poland (Dufresnes, Majtyka, et al., 2016). At contact, both species, whose homologous X and Y sex chromosomes are undifferentiated (Stöck et al., 2011, 2013), exhibit restricted introgression at sex‐linked compared to autosomal markers (Dufresnes, Majtyka, et al., 2016). Recent data from the Ukraine also suggest that both species also differ regarding their bioacoustics (Smirnov, 2013).

**FIGURE 1:**
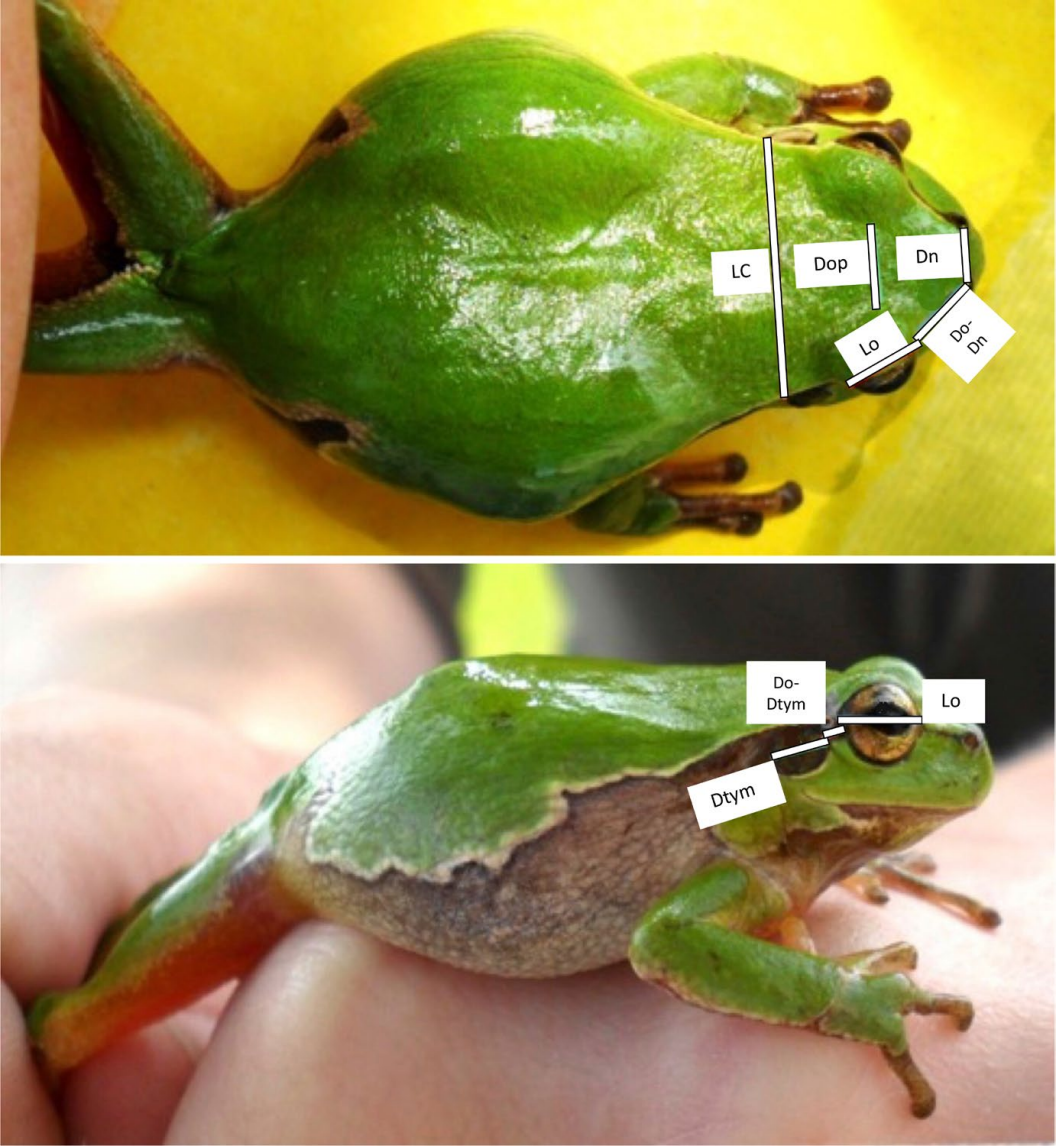
Measurements based on standardized photographs of tree frogs. (a) dorsal view; LC — width of the head, Dop—distance between the eyes, Do‐Dn — distance between the eye and the nostril, Dn — distance between the nostrils, Lo — eye diameter; (b) right side of the body; Lo — diameter of the eye, Ltym — diameter of the tympanum, Do‐Dtym — distance between the eye and the tympanum. See also Table 1 for abbreviations and definitions of all morphometric measurements

At first glance, *H*. *arborea* and *H*. *orientalis* appear morphologically virtually identical. They reach a snout‐vent‐length of about 5 cm, often have a lettuce‐green backside and a whitish to yellowish belly. Likely by similar physiological mechanisms as related tree frogs of the *Hyla japonica* group (Kang et al., 2016), both species are able to change their color and pattern against visually heterogeneous backgrounds. Between dorsal and ventral parts, usually from the nostrils to the after, runs a dark brown to black lateral stripe that is often bordered by a lighter upper margin and forms a characteristic sinus in the hip area (Figure 1).

Whereas genetic studies (Dufresnes, Litvinchuk, et al., 2016; Stöck et al., 2008, 2012) revealed two parapatric cryptic species in Poland, in the present paper, using a unique dataset of genotypically and morphometrically characterized *H*. *arborea* and *H*. *orientalis*, we focus on three major questions: (i) Do these species also differ in morphology? (ii) Are there morphometric differences between the parental species and their hybrids? And (iii) does hybridization lead to intermediate or transgressive morphotypes?

## 2 MATERIALS AND METHODS

### 2.1 Animal capture and genetic sampling

A total of 199 male tree frogs were caught during the breeding season 2011 (17 May–14 June), of which 191 were analyzed (see below). Tree frogs were caught at 23 sites, located in the central and northern part of Poland (see open access maps as “Figure 1” in: Dufresnes, Majtyka, et al., 2016). Because some morphological characters might be sexually dimorphic and since the usually much more cautiously behaving tree frog females are much harder to catch, morphological analyses were here restricted to males. Animals were caught at night with a dip net or manually in breeding ponds, using a flashlight. For genetic analyses, DNA samples (buccal swabs) were taken using cotton swabs (Broquet et al., 2007). Each frog was measured and photographed in a standardized manner (details below) at the collection site and then released immediately.

### 2.2 Genetic analysis

**FIGURE 2:**
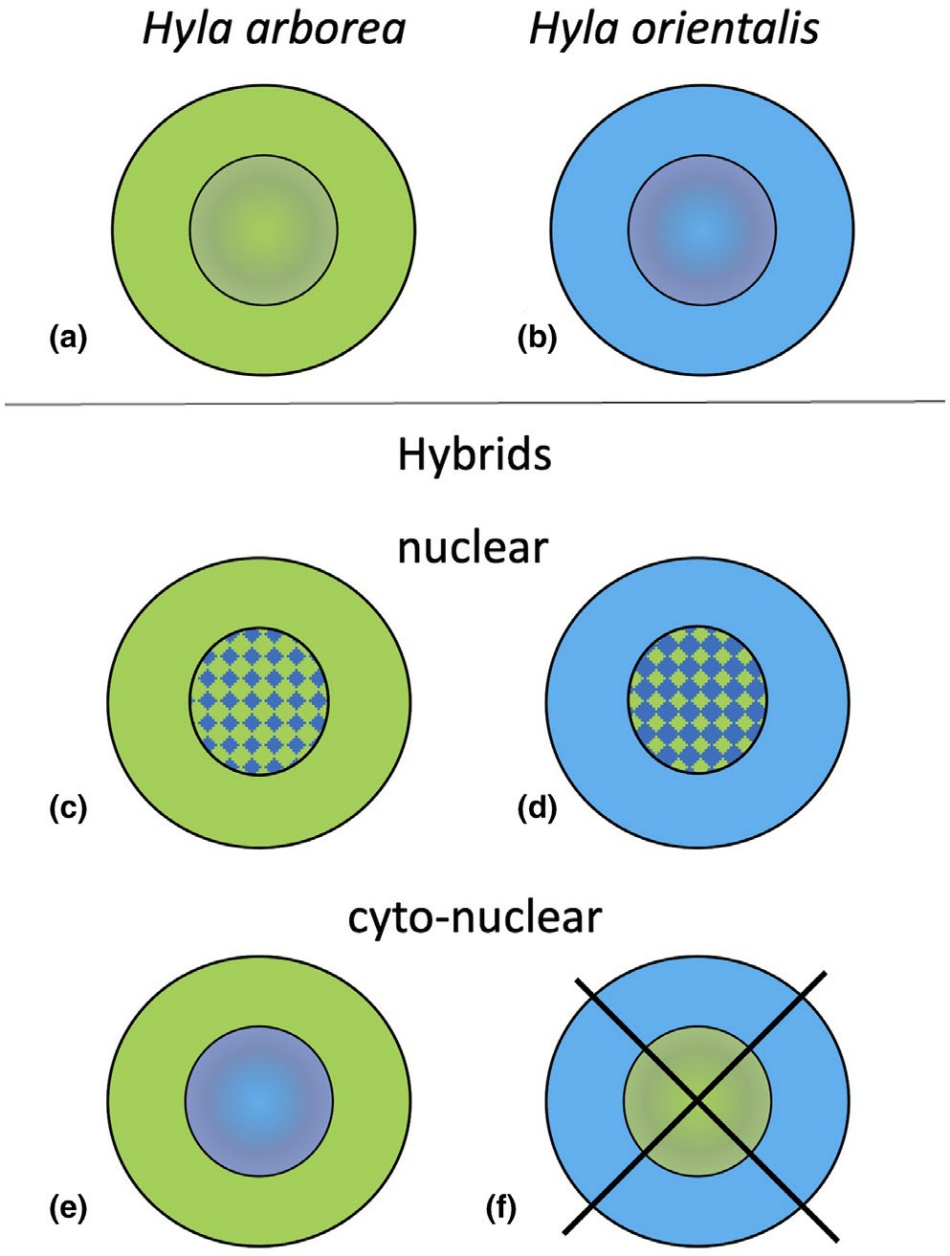
Simplified scheme of the genotypes of the parental species *Hyla arborea* and *H*. *orientalis* (a, b), and their different types of nuclear (c, d) and cyto‐nuclear hybrids (e, f). Parental species (a) *Hyla arborea*, (b) *H*. *orientalis*; (c) nuclear hybrid *H*. *arborea* x *H*. *orientalis* with mitochondrial (cytoplasmic) *H*. *arborea* genotype; (d) nuclear hybrid *H*. *orientalis* × *H*. *arborea* with mitochondrial (cytoplasmic) *H*. *orientalis* genotype; (e) cyto‐nuclear hybrid after multiple backcrosses with *H*. *orientalis* males and thus with mitochondrial (cytoplasmic) *H*. *arborea* genotype as the only detected traces of hybridity; (f) not detected, and therefore crossed out, in the study region: cyto‐nuclear hybrid after multiple backcrosses with *H*. *arborea* males and thus with mitochondrial (cytoplasmic) *H*. *orientalis* genotype as the only detected traces of hybridity

DNA from swabs was extracted using the DNeasy Blood and Tissue Kit (Qiagen, Hilden, Germany) using the protocol provided by the manufacturer for the Biosprint 96 (Qiagen) robot. The genotypes at 29 mainly transcriptome‐based microsatellite loci and the mtDNA haplotypes were assessed (for details on markers: Arens et al., 2000; Berset‐Brändli et al., 2008; Brelsford et al., 2013; Dufresnes et al., 2013; Dufresnes et al., 2014, 2014). Data on genetic analyses, including the male tree frogs studied here, have been published by Dufresnes, Majtyka, et al. (2016). Briefly, STRUCTURE v. 2.3.3 (Falush et al., 2003, 2007; Pritchard et al., 2000) served to detect patterns of genetic population structure. For the previously determined number of clusters (*K* = 2; Dufresnes, Majtyka, et al., 2016) and using default settings of the program (Pritchard et al., 2010), we ran ten simulations (algorithm Markov chain Monte Carlo, MCMC), each of which consisted of preliminary analysis (burn‐in) with 10,000 steps and 100,000 steps for the main analysis. Tree frogs were considered as “confirmed” nuclear hybrids between *H*. *arborea* and *H*. *orientalis* only, if 90% credible intervals (CIs) of their ancestry coefficient neither reached 0 nor 1. This conservative approach allows confidently assigned individuals to be distinguished from those with uninformative genotypes (Dufresnes, Majtyka, et al., 2016). As a result of repeated backcrossing and thereby introgression of mitochondrial DNA, some hybrid tree frogs exhibited a cyto‐nuclear discordance (equally termed in our paper “cyto‐nuclear hybrids”). These possessed mtDNA belonging to one species, in our case *H*. *arborea* but a nuclear assignment of *Q* ≥ 0.900 to *H*. *orientalis* (Figure 2; Stöck et al., 2021), and based on the available microsatellites could not necessarily be considered as nuclear hybrids. Therefore, these “cyto‐nuclear hybrids” may likewise not exhibit a hybrid morphological situation and thus were morphometrically also analyzed as a separate group, in several settings (see below and Stöck et al., 2021).

### 2.3 Morphometric measurements

Eleven (six direct and five image‐based) morphometric measurements were taken (for abbreviations and definitions: Table 1): six (SVL, LC, F, T, LP, LM) directly from the captured animals using an electric caliper (brand MAUa E1(VIS)) to an accuracy of 0.1 mm and five parameters (Do‐Dn, Dop, Lo, Ltym, Do‐Dtym) were measured using calibrated photographs (Figure 1). For this, standard images of each animal were taken from the dorsal and right sides from a distance of 20 cm. We then used Zeiss AxioVision software KS RUN 100 v. 3.0. To calibrate distances, we compared the width of the head (LC), taken directly from an animal, and compared it to the same distance on a photograph.

**TABLE 1:** Morphometric measurements with abbreviations as used in the text in alphabetic order and their definition, if required

Abbreviation	Measurement	Definition
Dn	distance between the nostrils	
Do‐Dn	distance between the eye and the nostril	straight distance from the front edges of the eye to the nostril
Do‐Dtym	distance between the eye and the tympanum	closest distance from the rear edge of the eye to the front edge of the tympanum
Dop	the distance between the eyes	distance between the nearest edges of the eyes
F	thigh (femur) length	distance from the after to the outer edge of the bent knee
LC	width of the head	straight distance from the rear edges of the left and right tympanum
LM	metatarsal length	distance from the edge of the heel bent to the base of the toe bent
Lo	diameter of the eye	
LP	foot length	distance from the outer edge of the bent heel to the end of the fourth toe
Ltym	diameter of the tympanum	
SVL	snout–vent length	straight distance from the end of the snout to the rear end of the cloaca
T	tibia length	distance from the outer edges of the bent knee to the bent heel

### 2.4 Statistical analyses

Initially, all data were tested for homogeneity of variances and normal distribution. For each morphometric parameter, we calculated mean, maximum and minimum, and standard deviation and performed a one‐way ANOVA and Tukey's multiple comparison tests (HSD). To test if groups could be separated without prior hypotheses on group membership, we conducted principal component analyses (PCA). PCAs were run on the correlation matrices. Individual principal components were considered as significant if their eigenvalues were equal or bigger than 1. Multivariate analyses of variance (MANOVA) served to check whether *H*. *arborea*, *H*. *orientalis* and/or hybrids’ PC scores differed. A Tukey post hoc test was then applied to test, which of the groups differed from each other. To find out if it is *a priori* possible analyzes.

### 2.5 Animals and groupings of parental species and hybrids

From the initially 199 male tree frogs caught, 8 exhibited uninformative genotypes (in 2 males mtDNA could not be amplified; in 6 potential nuclear hybrids 90% credible intervals of their ancestry coefficients reached 0 or 1 and thus were not confirmed; cf. Dufresnes, Majtyka, et al., 2016). Thus, the full dataset comprised 191 male frogs. Analysis of 29 microsatellite loci and mtDNA haplotypes revealed 72 *H*. *arborea*, 66 *H*. *orientalis* (Figure 2a and b), and 53 hybrids (Figure 2c–e). All hybrids (from now: “pooled hybrids”; Stöck et al., 2021) exhibited assignment probabilities with various mitochondrial and nuclear‐DNA‐based signals of genetic admixture: 29 frogs were assigned as hybrids based on nuclear DNA markers (from now: “nuclear hybrids,” Figure 2c and d), 24 could only be identified due to mtDNA‐introgression (from now: “cyto‐nuclear hybrids”) with *H*. *arborea* mtDNA and *H*. *orientalis* nuclear DNA (as in Figure 2c); the reciprocal combination (Figure 2f) was not found in our sample, and 8 were subadult specimens < 39 mm (SVL) and thus were only included in analyses with pooled hybrids (see below).

To better understand how hybrid status as well as body size translate into potential morphometric differences, we analyzed the data in three different groupings: (i), including pure *H*. *arborea*, pure *H*. *orientalis*, and pooled hybrids (i.e., both those based on nuclear microsatellites and those only exhibiting mtDNA introgression); (ii), as above, but all individuals < 39 mm were left out, because they present immature juveniles, in which the adult morphotype might not be completely established (see also Discussion on allometry); and (iii), as in (ii) but further subdivided the hybrids into two sub‐groups: those with a clear signature of nuclear hybridization (nuclear hybrids as in Figure 2c and d) and those only detected based on mitochondrial DNA introgression (cyto‐nuclear hybrids as in Figure 2e).

## 3 RESULTS

### 3.1 Descriptive statistics and one‐way ANOVAs

Comparisons between *H*. *arborea*, *H*. *orientalis* and their hybrids revealed significant differences between the means of many of the parameters as calculated for each of the three groupings (i–iii; Table S1a‐c). Specifically, all types of hybrids (grouping (i)) differed from *H*. *arborea* for 6 characters: width of the head, length of femur, diameter of the tympanum, distances between the nostrils and the eye, and distance between the nostrils (LC, F, LTym, Do‐Dn, and Dn), but only for three from *H*. *orientalis*: tibia length, foot length and distance between nostrils (T, LP, Dn) and this bias increased for adult hybrids (> 39 mm; grouping (ii)). Finally, for grouping (iii) with a subdivision of hybrids, nuclear hybrids differed from *H*. *arborea* in 5 characters: femur length, foot length, distance between eye and nostril (F, LP, Do‐Dn, Dn and Lo) but only for one (T) from *H*. *orientalis*, while cyto‐nuclear hybrids showed differences from *H*. *arborea* in five characters: width of head, foot length, diameter of the tympanum, and distance between the nostrils (LC, LP, Ltym, Do‐Dn, and Dn) but only for one (T) from *H*. *orientalis*.

In summary, while *H*. *arborea* and *H*. *orientalis* appeared to differ between each other in several parameters, hybrids tended to show more overlap in morphology with *H*. *orientalis* than with *H*. *arborea*. The parameter length of tibia (T) differentiated all types of hybrids from *H*. *orientalis*, while Do‐Dn distinguished them from *H*. *arborea*.

### 3.2 Multivariate analyses

Principal component analyses (PCA) in pure species and hybrids were conducted to reveal potential internal structure of the data that best explain their variance and to produce a lower dimensional projection from the most informative viewpoint.

**FIGURE 3:**
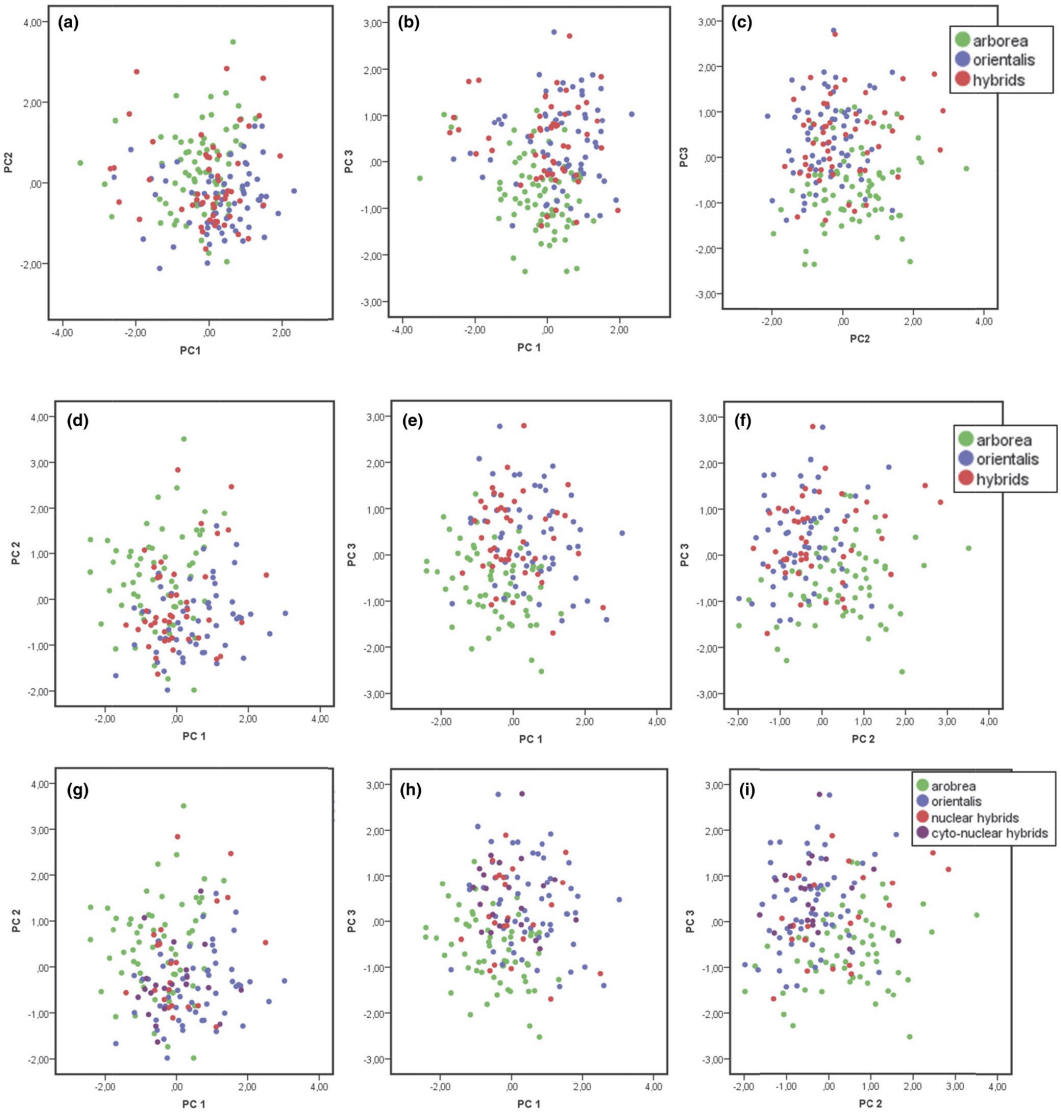
Results of principal component analyses (PCAs) for *Hyla arborea*, *H*. *orientalis*, and their hybrids (groupings i to iii; see main text for further explanation); shown are axes 1–3. (a–c) Grouping (i), all individuals, pooled hybrids (i.e., both, those based on nuclear microsatellites and those only exhibiting mtDNA‐introgression); (d–f) Grouping (ii), only including tree frogs >39 mm (SVL), pooled hybrids; (g–i) Grouping (iii), only including tree frogs > 39 mm (SVL), subdivided hybrids as shown by color coding

For grouping (i), PCA for all specimens yielded three components with eigenvalues > 1 (Figure 3a–c; Tables S2a and S3a), providing significant differences between *H*. *orientalis* and *H*. *arborea* along the 1st (strongly loading parameters: SVL, LC, LM, T, F, LP; Table 1) and 2nd axes (strongly loading: Ltym, Do‐Dtym), and less pronounced differences between each of the two species and the group of pooled hybrids along the 2nd and 3rd axes (strongly loading: Do‐Dn).

A PCA for specimens > 39 mm (grouping ii) yielded three components with eigenvalues > 1 (Figure 3d‐f, Tables S2b and S3b), where differences arose between both parental species and each of them to the pooled hybrids along the 1st axis (strongly loading: SVL, T, F, LP) and between *H*. *orientalis* and *H*. *arborea* and the latter to the pooled hybrids along the 2nd (Ltym, Do‐Dtym, Do‐Dn) and the 3rd axes (no strongly loading parameters).

**FIGURE 4:**
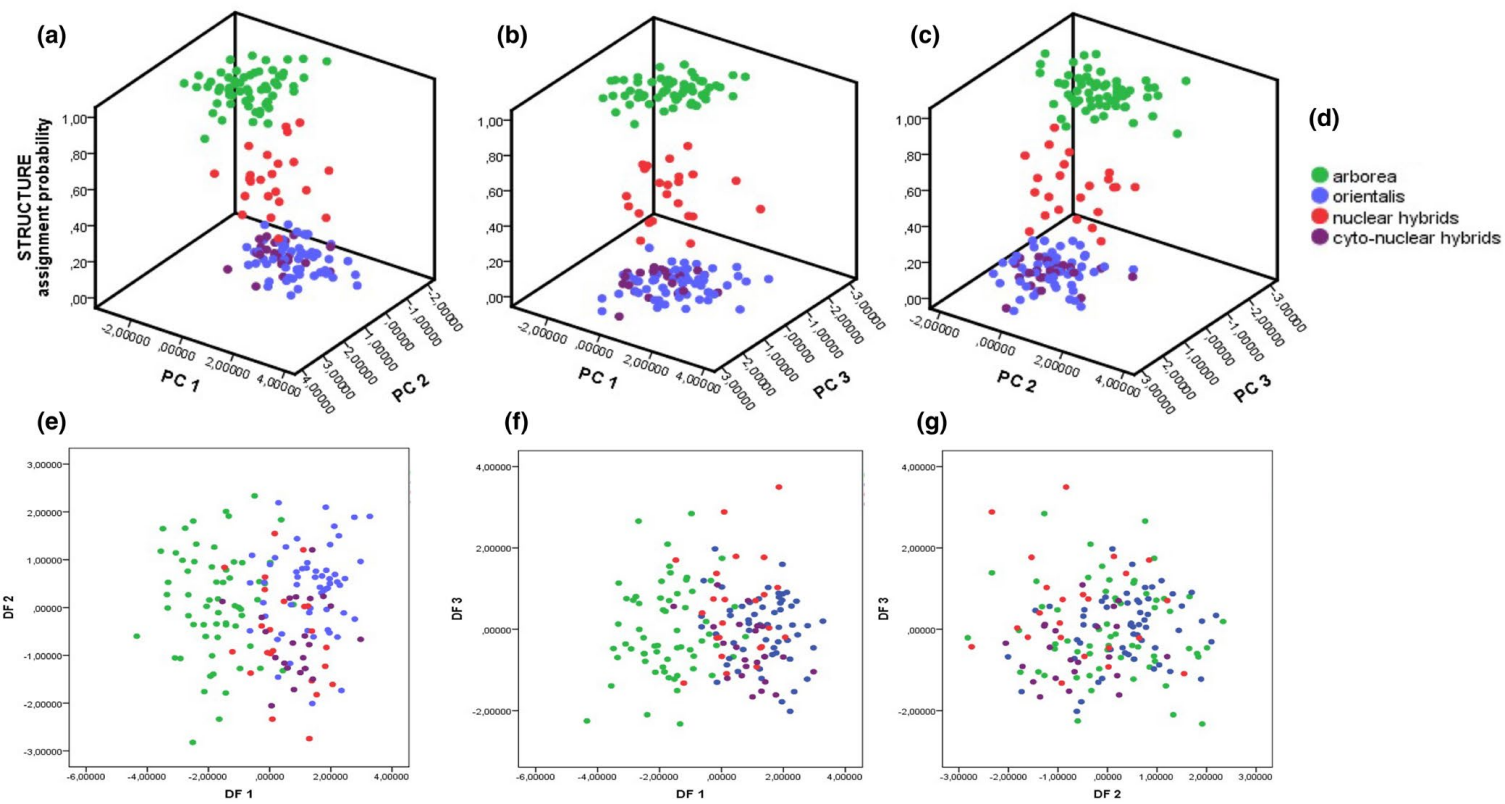
Multivariate analyses (PCAs, DFAs) for *Hyla arborea*, *H*. *orientalis*, and their nuclear and cyto‐nuclear hybrids (grouping iii; main text for further explanation). (a–c) Results of a principal component analysis (PCA) in comparison with assignment probabilities from the program STRUCTURE, based on genotyping with 27 nuclear microsatellites; comparing axes 1–3. (d) Color‐coding of the four groups of tree frogs. (e–g) Results of a discriminant function analysis (DFA); comparing axes 1–3

This same PCA (Figure 3g–i; Tables S2c and S3c), applied to the grouping (iii) with subdivided hybrids, led again to clear differences between *H*. *orientalis* and *H*. *arborea* as well as between the latter and the nuclear hybrids along the 1st axis (identical strongly loading parameters: SVL, T, F, LP). Differences were also revealed between both parental species as well as between *H*. *arborea* and the cyto‐nuclear hybrids along the 2nd axis (Ltym, Do‐Dtym, Do‐Dn) and finally between both parental species and *H*. *arborea* and the nuclear and cyto‐nuclear hybrids along the 3rd axis. Figure 4a–c show results of a principal component analysis (PCA) for grouping iii in comparison with assignment probabilities from the program STRUCTURE, based on genotyping with 27 nuclear microsatellites; comparing axes 1–3.

Taken together, all PCAs revealed internal structure for the external features of these tree frogs and demonstrate clear morphological differences among both parental species and pooled as well as subdivided hybrids, with the latter being easier distinguishable from *H*. *arborea* than from *H*. *orientalis*.

Discriminant function analyses (DFAs) confirmed significant differences between most groups of both parental species and pooled (grouping i and ii; Figures S1a, b; Tables S4a, b and S5a, b) as well as subdivided hybrids (grouping iii: Tables S4c and S5c, Figure 4e‐g). Although for grouping (i), *H*. *orientalis* were not distinguishable from pooled hybrids, specimens > 39 mm (grouping ii) could be well discriminated.

Groupwise re‐classification scores for DFAs (Tables S5a–c) were generally high for pure species, with the highest for *H*. *arborea* (83–85%), distinctly lower for *H*. *orientalis* (66%–73%) and lower scores (60%–68%) for pooled hybrids (groupings i and ii) and the lowest for subdivided hybrids (63% cyto‐nuclear and 52% for nuclear hybrids, grouping iii).

Importantly, pooled hybrids were mostly (24%) re‐classified as *H*. *orientalis* but much less (6%–15%) into *H*. *arborea* (groupings i, ii). Likewise, subdivided hybrids (grouping iii) were re‐classified considerably into *H*. *orientalis* (17%–18% for nuclear and cyto‐nuclear hybrids) than into *H*. *arborea* (9%–4%), respectively. DFAs calculated based on parental species only likewise led to the reclassification of a majority of hybrids into *H*. *orientalis* (Tables S6a–c).

## 4 DISCUSSION

In the contact and hybrid zone of two Eastern‐European hylids, we have morphometrically examined genotyped male tree frogs and their natural hybrids. Our analyses showed the morphotypes of both parental species (*H*. *arborea*, *H*. *orientalis*) and their various (pooled or subdivided) hybrids to differ, with the hybrid morphology tending to be more similar to *H*. *orientalis* than to *H*. *arborea*. Despite some potential influence of known allometric ontogenetic changes in anurans as well as in *Hyla* (Shrimpton et al., 2021), our tests involving only larger‐sized adults made it improbable that this phenomenon had a major relevant impact on our results.

Univariate comparisons of both cryptic species revealed differences of the means of many morphometric characters between the parental forms as well as between their hybrids, suggesting that these, for the unaided eye, identical morphotypes in fact present not only genetically but also morphologically distinct forms. For several parameters, means of hybrids showed intermediate values between the pure species but a majority of hybrid characters overlapped with *H*. *orientalis*. These results were confirmed for all three groupings (i–iii) using multivariate exploratory methods (PCA) as well as techniques with prior grouping (DFA). Likewise, for DFAs based on parental species only, re‐assignment of hybrids showed them to be more likely to be classified as *H*. *orientalis* than as *H*. *arborea* (Tables S6a–c).

In conclusion, multiple lines of evidence suggest that hybrid morphotypes are neither intermediate between the parental species nor beyond the range of their morphological variation, and thus, the generation of transgressive morphotypes by hybridization of *H*. *orientalis* and *H*. *arborea* can be rejected (at least for males).

### 4.1 Expectations and preconditions for evolution of transgressive phenotypes in the study system

Transgressive segregation requires quantitative trait loci with antagonistic effects (opposite to the direction of mean phenotypic variances) in the parental populations (Albertson & Kocher, 2005; Rieseberg et al., 2003). Stabilizing selection, genetic drift, or varying selective regimes in the evolutionary history may promote the evolution of transgressive loci, while consistent selective regimes may favor the accumulation of alleles with steady effects (Albertson & Kocher, 2005; Orr, 1998). At immediate contact, epigenetic factors may be contributing to the occurrence and thus evolution of transgressive patterns seen in hybridizing lineages (Cooper & Shaffer, 2021 incl. refs.).

Due to the relatively well‐documented complex phylogeography of the tree frogs examined (see Introduction) and thus widely different evolutionary histories (e.g., comprising different glacial refugia, specific routes of postglacial re‐colonization, and very unequal intraspecific genetic diversities) of *H*. *orientalis* and *H*. *arborea* let us assume genomic potential for the transgressive segregation. While our study rejects the occurrence of transgressive hybrid morphotypes, we are aware of the limitations of our work. Our inference is limited to relatively few morphometric characters as well as to only male frogs. Thus, we cannot exclude the evolution of female transgressive morphologies and generally have at present no information about any other phenotypic traits such as ecological niches, disease resistance, and behavior. that might well show hybrid transgression in our target species.

### 4.2 Remarks on the distinguishability of the cryptic species *H. orientalis* and *H. arborea* in the field

Morphological criteria alone may be, obviously, often be misleading reading the hybrid status of particular individuals (e.g., Babik et al., 2003; Lamb & Avise, 1987). Our data set allowed comparisons between genotypes and morphotypes. We found that *H*. *arborea*, *H*. *orientalis*, and their hybrids differ in morphometry with the hybrids exhibiting greater similarity to *H*. *orientalis*. For field studies, due to widely overlapping ranges of parameters (Table S1a–c), we here cannot provide single morphometric traits for discrimination. A similar situation was reported by Bruschi et al. (2006), who compared seven morphometric traits in *H*. *sarda* and *H*. *meridionalis*, two species of the *Hyla arborea* group from Italy. Such for the human perception hardly accessible taxa, sibling or cryptic species (e.g., Fišer et al., 2018), were often first identified by molecular approaches for species recognition. Based on morphological and especially coloration characters in Ukraine and Romania, Bedriaga (1890) distinguished four tree frog varieties including “*H*. *arborea* Typus” and “*H*. *a*. Var. *orientalis*,” the latter soon being synonymized with the former (Boulenger, 1898).

However, Bedriaga (1890), describing the two forms as the “varieties” *orientalis* and *arborea*, likewise found nearly no morphometric differences. He stated that foot length (LP) is roughly equal to the length of tibia (T); also tibia (T) and femur (F) were of similar lengths. The ratio of foot to tibia length (F/T) by Bedriaga (1890) could not be directly compared to our results since Bedriaga (1890) measured the length of the foot as the distance from the *callus internus* to the tip of the longest toe, while we measured the distance from the heel.

## 5 CONCLUSIONS

While we have shown here that hybrid morphotypes neither exhibit intermediacy nor transgression but appear biased to *H*. *orientalis*, other phenotypic properties, reaching from ecology to behavior, remain to be examined. Anuran radiations, like the Western Palearctic tree frogs, with a variety of lineages of different divergence time, and multiple hybrid zones bear a great potential to study morphological and phenotypic evolution more thoroughly along with genetic data.

## CONFLICT OF INTEREST

None.

## AUTHOR CONTRIBUTIONS

**Tomasz Majtyka:** Conceptualization (leading); Data curation (supporting); Formal analysis (supporting); Investigation (equal); Methodology (equal); Writing – original draft (leading); Writing – review & editing (supporting). **Bartosz Borczyk:** Data curation (supporting); Formal analysis (lead); Writing – review & editing (supporting). **Maria Ogielska:** Conceptualization (supporting); Data curation (equal); Funding acquisition (equal); Project administration (equal); Supervision (lead); Visualization (equal); Writing – original draft (equal); Writing – review & editing (equal). **Matthias Stöck:** Conceptualization (equal); Data curation (equal); Formal analysis (equal); Investigation (equal); Methodology (equal); Validation (equal); Visualization (equal); Writing – original draft (equal); Writing – review & editing (equal).

## Supporting information

Supplementary Figure S1; Supplementary Tables S1‐S6.

## Data Availability

Morphometric data for all parameters (as shown in Table 1) for each individual frog along with STRUCTURE‐based assignments (Q) from nuclear DNA‐based microsatellite genotypes to either *H*. *arborea* or H. *orientalis*, their mitochondrial DNA genotype and the numbers examined from both parental species and the different types of hybrids (nuclear, cyto‐nuclear, pooled) are available from Dryad (Stöck et al., 2021, https://doi.org/10.5061/dryad.mkkwh7122).
