# Supplementary material for: Morphometry of two cryptic tree frog species at their hybrid zone reveals neither intermediate nor transgressive morphotypes
Source: Ecol Evol. 2022 Jan 27;12(1):e8527. doi: 10.1002/ece3.8527 (PMC8794711; doi:10.1002/ece3.8527)
Supplement: Supplementary file 1 — Supplementary Figure S1; Supplementary Tables S1‐S6. [file ECE3-12-e8527-s001.docx]

Supporting Information for:

**Morphometry of two cryptic tree frog species at their hybrid zone reveals neither intermediate nor transgressive morphotypes**

**Authors: Tomasz Majtyka, Bartosz Borczyk, Maria Ogielska, Matthias Stöck**

**FIGURE S1 Results of a discriminant function analysis (DFA);**

**comparing axes 1 and 2 for *Hyla arborea, H. orientalis*, and their pooled hybrids.**

**(a)** Grouping i: for all tree frogs examined,

**(b)** Grouping ii: only including tree frogs > 39 mm (SVL)


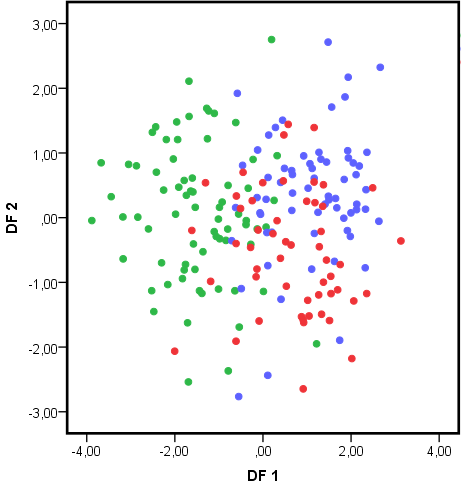

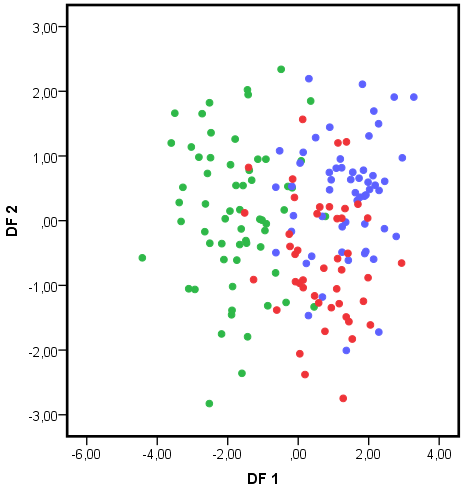


a

b


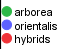


**TABLE S1a One-way ANOVA and descriptive statistics (grouping i)**

(1: *H. arborea,* N = 72; 2: *H. orientalis,* N = 66; 3: pooled hybrids, N = 53)

|  | | min | max | mean | ±SD | F | *P* | *Posthoc* | *p* |
| --- | --- | --- | --- | --- | --- | --- | --- | --- | --- |
| SVL | *H. arborea* | 32.10 | 46.20 | 41.0681 | ±2.80044 | 1.12 | 0.329 | *orientalis* | 0.310 |
|  | *H. orientalis* | 34.50 | 47.30 | 41.8030 | ±2.82870 |  |  | p. hybrids | 0.594 |
|  | pooled hybrids | 32.40 | 47.30 | 41.2736 | ±3.26244 |  |  | *arborea* | 0.921 |
| LC | *H. arborea* | 11.20 | 16.40 | 13.6750 | ±0.91955 | 3.137 | 0.046 | *orientalis* | 0.841 |
|  | *H. orientalis* | 11.40 | 15.50 | 13.5833 | ±0.95621 |  |  | p. hybrids | 0.151 |
|  | pooled hybrids | 10.40 | 15.60 | 13.2528 | ±1.01368 |  |  | *arborea* | 0.042 |
| LM | *H. arborea* | 6.80 | 12.00 | 9.9444 | ±0.95341 | 3.139 | 0.046 | *orientalis* | 0.065 |
|  | *H. orientalis* | 7.60 | 12.80 | 10.2955 | ±0.86102 |  |  | p. hybrids | 0.105 |
|  | pooled hybrids | 7.40 | 11.70 | 9.9509 | ±0.92167 |  |  | *arborea* | 0.999 |
| T | *H. arborea* | 13.90 | 22.20 | 18.9472 | ±1.58247 | 8.931 | < 0.001 | *orientalis* | <0.001 |
|  | *H. orientalis* | 15.50 | 22.90 | 20.0712 | ±1.59757 |  |  | p. hybrids | 0.015 |
|  | pooled hybrids | 14.80 | 22.00 | 19.2434 | ±1.62700 |  |  | *arborea* | 0.563 |
| F | *H. arborea* | 13.10 | 24.10 | 19.5375 | ±2.34331 | 9.612 | < 0.001 | *orientalis* | <0.001 |
|  | *H. orientalis* | 16.20 | 25.40 | 21.0530 | ±1.77315 |  |  | p. hybrids | 0.052 |
|  | pooled hybrids | 15.10 | 23.90 | 20.1736 | ±1.87167 |  |  | *arborea* | 0.197 |
| LP | *H. arborea* | 22.50 | 34.50 | 29.8986 | ±2.54511 | 13.185 | < 0.001 | *orientalis* | <0.001 |
|  | *H. orientalis* | 23.90 | 37.20 | 32.4758 | ±2.60308 |  |  | p. hybrids | 0.03 |
|  | pooled hybrids | 12.30 | 38.10 | 31.0830 | ±3.74888 |  |  | *arborea* | 0.07 |
| LTym | *H. arborea* | 2.00 | 4.30 | 2.9403 | ±0.44553 | 6.08 | 0.003 | *orientalis* | 0.014 |
|  | *H. orientalis* | 1.80 | 3.60 | 2.7333 | ±0.42655 |  |  | p. hybrids | 0.907 |
|  | pooled hybrids | 1.70 | 3.60 | 2.7000 | ±0.40903 |  |  | *arborea* | 0.006 |
| Do_Dtym | *H. arborea* | 1.00 | 2.10 | 1.5056 | ±0.28428 | 4.908 | 0.008 | *orientalis* | 0.007 |
|  | *H. orientalis* | 0.80 | 2.20 | 1.3530 | ±0.27187 |  |  | p. hybrids | 0.760 |
|  | pooled hybrids | 0.90 | 2.40 | 1.4679 | ±0.33153 |  |  | *arborea* | 0.089 |
| Do_Dn | *H. arborea* | 1.30 | 3.00 | 2.2917 | ±0.37105 | 36.0.59 | < 0.001 | *orientalis* | <0.001 |
|  | *H. orientalis* | 1.80 | 3.90 | 2.8273 | ±0.42951 |  |  | p. hybrids | 0.520 |
|  | pooled hybrids | 2.10 | 3.70 | 2.7472 | ±0.39106 |  |  | *arborea* | <0.001 |
| Dop | *H. arborea* | 3.20 | 4.50 | 3.9403 | ±0.32400 | 3.822 | 0.024 | *orientalis* | 0.035 |
|  | *H. orientalis* | 2.90 | 5.20 | 4.1136 | ±0.44303 |  |  | p. hybrids | 0.079 |
|  | pooled hybrids | 3.30 | 5.20 | 4.1000 | ±0.45573 |  |  | *arborea* | 0.982 |
| Dn | *H. arborea* | 2.00 | 3.20 | 2.7514 | ±0.27062 | 17.425 | < 0.001 | *orientalis* | <0.001 |
|  | *H. orientalis* | 2.30 | 3.60 | 3.0485 | ±0.32734 |  |  | p. hybrids | 0.041 |
|  | pooled hybrids | 2.10 | 3.50 | 2.9151 | ±0.28849 |  |  | *arborea* | 0.007 |
| Lo | *H. arborea* | 2.00 | 3.60 | 2.8995 | ±0.32077 | 2.091 | 0.126 | *orientalis* | 0.190 |
|  | *H. orientalis* | 2.80 | 5.10 | 4.1924 | ±0.44766 |  |  | p. hybrids | 0.996 |
|  | pooled hybrids | 3.20 | 5.70 | 4.2000 | ±0.47515 |  |  | *arborea* | 0.195 |

**TABLE S1b One-way ANOVA and descriptive statistics (grouping ii)**

(1: *H. arborea*, N = 61; 2: *H. orientalis*, N = 56; 3: hybrids, N = 45)

|  | | min | max | mean | ±SD | F | p | *Posthoc* | *p* |
| --- | --- | --- | --- | --- | --- | --- | --- | --- | --- |
| SVL | *H. arborea* | 39.10 | 46.20 | 41.9393 | ±1.73535 | 2.262 | 0.107 | *orientalis* | 0.093 |
|  | *H. orientalis* | 39.40 | 47.30 | 42.6696 | ±2.02950 |  |  | p. hybrids | 0.754 |
|  | pooled hybrids | 39.20 | 47.30 | 42.4000 | ±1.87192 |  |  | *arborea* | 0.427 |
| LC | *H. arborea* | 12.20 | 16.40 | 13.8951 | ±0.75109 | 3.226 | 0.042 | *orientalis* | 0.954 |
|  | *H. orientalis* | 12.50 | 15.50 | 13.8554 | ±0.73951 |  |  | p. hybrids | 0.099 |
|  | pooled hybrids | 12.30 | 15.60 | 13.5511 | ±0.70376 |  |  | *arborea* | 0.427 |
| LM | *H. arborea* | 6.80 | 12.00 | 10.0918 | ±0.84996 | 5.76 | 0.004 | *orientalis* | 0.003 |
|  | *H. orientalis* | 9.40 | 12.80 | 10.5375 | ±0.61602 |  |  | p. hybrids | 0.096 |
|  | pooled hybrids | 8.80 | 11.70 | 10.2378 | ±0.63721 |  |  | *arborea* | 0.557 |
| T | *H. arborea* | 17.10 | 22.20 | 19.3279 | ±1.15226 | 19.785 | < 0.001 | *orientalis* | <0.001 |
|  | *H. orientalis* | 18.20 | 22.90 | 20.5554 | ±1.08777 |  |  | p. hybrids | 0.002 |
|  | pooled hybrids | 18.60 | 22.00 | 19.8244 | ±0.86738 |  |  | *arborea* | 0.047 |
| F | *H. arborea* | 14.70 | 24.10 | 19.9459 | ±1.99203 | 15.847 | < 0.001 | *orientalis* | <0.001 |
|  | *H. orientalis* | 18.90 | 25.40 | 21.5625 | ±1.32577 |  |  | p. hybrids | 0.002 |
|  | pooled hybrids | 18.80 | 23.90 | 20.8289 | ±1.06571 |  |  | *arborea* | 0.047 |
| LP | *H. arborea* | 27.50 | 34.50 | 30.5328 | ±1.86322 | 33.861 | 0.001 | *orientalis* | <0.001 |
|  | *H. orientalis* | 28.90 | 37.20 | 33.2125 | ±1.88854 |  |  | p. hybrids | 0.037 |
|  | pooled hybrids | 30.00 | 38.10 | 32.3200 | ±1.56097 |  |  | *arborea* | <0.001 |
| LTym | *H. arborea* | 2.40 | 4.30 | 3.0213 | ±0.40046 | 7.350 | 0.001 | *orientalis* | 0.007 |
|  | *H. orientalis* | 2.10 | 3.60 | 2.7929 | ±0.41510 |  |  | p. hybrids | 0.849 |
|  | pooled hybrids | 2.20 | 3.60 | 2.7489 | ±0.39117 |  |  | *arborea* | 0.002 |
| Do_Dtym | *H. arborea* | 1.00 | 2.00 | 1.5279 | ±0.27394 | 5.69 | 0.004 | *orientalis* | 0.003 |
|  | *H. orientalis* | 1.00 | 2.20 | 1.3518 | ±0.26215 |  |  | p. hybrids | 0.104 |
|  | pooled hybrids | 1.00 | 2.40 | 1.4689 | ±0.32531 |  |  | *arborea* | 0.545 |
| Do_Dn | *H. arborea* | 1.30 | 3.00 | 2.3295 | ±0.37164 | 33.001 | < 0.001 | *orientalis* | <0.001 |
|  | *H. orientalis* | 2.10 | 3.90 | 2.8839 | ±0.41331 |  |  | p. hybrids | 0.450 |
|  | pooled hybrids | 2.10 | 3.70 | 2.7889 | ±0.39556 |  |  | *arborea* | <0.001 |
| Dop | *H. arborea* | 3.20 | 4.50 | 3.9770 | ±0.32062 | 4.251 | 0.016 | *orientalis* | 0.030 |
|  | *H. orientalis* | 2.90 | 5.20 | 4.1696 | ±0.45163 |  |  | p. hybrids | 0.999 |
|  | pooled hybrids | 3.40 | 5.20 | 4.1667 | ±0.44313 |  |  | *arborea* | 0.048 |
| Dn | *H. arborea* | 2.00 | 3.20 | 2.7705 | ±0.27285 | 26.255 | < 0.001 | *orientalis* | <0.001 |
|  | *H. orientalis* | 2.60 | 3.60 | 3.1250 | ±0.26985 |  |  | p. hybrids | 0.017 |
|  | pooled hybrids | 2.40 | 3.50 | 2.9778 | ±0.25126 |  |  | *arborea* | <0.001 |
| Lo | *H. arborea* | 3.30 | 6.20 | 4.1164 | ±0.50437 | 2.616 | 0.076 | *orientalis* | 0.093 |
|  | *H. orientalis* | 3.40 | 5.10 | 4.2911 | ±0.37575 |  |  | p. hybrids | 0.973 |
|  | pooled hybrids | 3.80 | 5.70 | 4.2711 | ±0.45308 |  |  | *arborea* | 0.189 |

**TABLE S1c One-way ANOVA and descriptive statistics (grouping iii)**

(1: *H. arborea*, N = 61, 2: *H. orientalis,* N = 56; 3: nuclear hybrids, N = 23; 4: cytonuclear hybrids, N = 22).

|  | | min | max | mean | SD | F | p | *Posthoc* | *p* |
| --- | --- | --- | --- | --- | --- | --- | --- | --- | --- |
| SVL | *H. arborea* | 39.10 | 46.20 | 41.9393 | 1.73535 | 1.658 | 0.178 | *orientalis* | 0.159 |
|  | *H. orientalis* | 39.40 | 47.30 | 42.6696 | 2.02950 |  |  | cyto-nuclear hybrids | 0.760 |
|  | cyto-nuclear hybrids | 39.20 | 47.30 | 42.5870 | 2.04679 |  |  | *arborea* | 0.942 |
|  | nuclear hybrids | 39.70 | 45.60 | 42.2045 | 1.69551 |  |  | *arborea* | 0.497 |
|  | cyto-nuclear hybrids |  |  |  |  |  |  | *orientalis* | 0.760 |
|  | nuclear hybrids |  |  |  |  |  |  | cyto-nuclear hybrids | 0.904 |
| LC | *H. arborea* | 12.20 | 16.40 | 13.8951 | 0.75109 | 2.628 | 0.052 | *orientalis* | 0.991 |
|  | *H. orientalis* | 12.50 | 15.50 | 13.8554 | 0.73951 |  |  | cyto-nuclear hybrids | 0.087 |
|  | cyto-nuclear hybrids | 12.60 | 15.60 | 13.6783 | 0.61493 |  |  | *arborea* | 0.048 |
|  | nuclear hybrids | 12.30 | 14.90 | 13.4182 | 0.77805 |  |  | *arborea* | 0.622 |
|  | cyto-nuclear hybrids |  |  |  |  |  |  | *orientalis* | 0.087 |
|  | nuclear hybrids |  |  |  |  |  |  | cyto-nuclear hybrids | 0.634 |
| LM | *H. arborea* | 6.80 | 12.00 | 10.0918 | 0.84996 | 4.161 | 0.007 | *orientalis* | 0.006 |
|  | *H. orientalis* | 9.40 | 12.80 | 10.5375 | 0.61602 |  |  | cyto-nuclear hybrids | 0.713 |
|  | cyto-nuclear hybrids | 8.80 | 11.70 | 10.1348 | 0.70040 |  |  | *arborea* | 0.489 |
|  | nuclear hybrids | 9.10 | 11.20 | 10.3455 | 0.55953 |  |  | *arborea* | 0.995 |
|  | cyto-nuclear hybrids |  |  |  |  |  |  | *orientalis* | 0.713 |
|  | nuclear hybrids |  |  |  |  |  |  | cyto-nuclear hybrids | 0.759 |
| T | *H. arborea* | 17.10 | 22.20 | 19.3279 | 1.15226 | 13.143 | < 0.001 | *orientalis* | 0.000 |
|  | *H. orientalis* | 18.20 | 22.90 | 20.5554 | 1.08777 |  |  | cyto-nuclear hybrids | 0.021 |
|  | cyto-nuclear hybrids | 18.80 | 22.00 | 19.8696 | 0.95700 |  |  | *arborea* | 0.325 |
|  | nuclear hybrids | 18.60 | 21.70 | 19.7773 | 0.78251 |  |  | *arborea* | 0.162 |
|  | cyto-nuclear hybrids |  |  |  |  |  |  | *orientalis* | 0.021 |
|  | nuclear hybrids |  |  |  |  |  |  | cyto-nuclear hybrids | 0.991 |
| F | *H. arborea* | 14.70 | 24.10 | 19.9459 | 1.99203 | 10.592 | < 0.001 | *orientalis* | 0.000 |
|  | *H. orientalis* | 18.90 | 25.40 | 21.5625 | 1.32577 |  |  | cyto-nuclear hybrids | 0.138 |
|  | cyto-nuclear hybrids | 19.40 | 23.90 | 20.9391 | 1.08449 |  |  | *arborea* | 0.200 |
|  | nuclear hybrids | 18.80 | 22.80 | 20.7136 | 1.05843 |  |  | *arborea* | 0.049 |
|  | cyto-nuclear hybrids |  |  |  |  |  |  | *orientalis* | 0.138 |
|  | nuclear hybrids |  |  |  |  |  |  | cyto-nuclear hybrids | 0.962 |
| LP | *H. arborea* | 27.50 | 34.50 | 30.5328 | 1.86322 | 22.516 | < 0.001 | *orientalis* | 0.000 |
|  | *H. orientalis* | 28.90 | 37.20 | 33.2125 | 1.88854 |  |  | cyto-nuclear hybrids | 0.120 |
|  | cyto-nuclear hybrids | 30.00 | 38.10 | 32.4304 | 1.87385 |  |  | *arborea* | 0.001 |
|  | nuclear hybrids | 30.30 | 35.60 | 32.2045 | 1.18301 |  |  | *arborea* | 0.000 |
|  | cyto-nuclear hybrids |  |  |  |  |  |  | *orientalis* | 0.120 |
|  | nuclear hybrids |  |  |  |  |  |  | cyto-nuclear hybrids | 0.975 |
| LTym | *H. arborea* | 2.40 | 4.30 | 3.0213 | 0.40046 | 5.653 | 0.001 | *orientalis* | 0.013 |
|  | *H. orientalis* | 2.10 | 3.60 | 2.7929 | 0.41510 |  |  | cyto-nuclear hybrids | 0.549 |
|  | cyto-nuclear hybrids | 2.20 | 3.60 | 2.8348 | 0.41627 |  |  | *arborea* | 0.002 |
|  | nuclear hybrids | 2.20 | 3.60 | 2.6591 | 0.35005 |  |  | *arborea* | 0.223 |
|  | cyto-nuclear hybrids |  |  |  |  |  |  | *orientalis* | 0.549 |
|  | nuclear hybrids |  |  |  |  |  |  | cyto-nuclear hybrids | 0.460 |
| Do_Dtym | *H. arborea* | 1.00 | 2.00 | 1.5279 | 0.27394 | 4.449 | 0.005 | *orientalis* | 0.006 |
|  | *H. orientalis* | 1.00 | 2.20 | 1.3518 | 0.26215 |  |  | cyto-nuclear hybrids | 0.854 |
|  | cyto-nuclear hybrids | 1.00 | 2.40 | 1.5261 | 0.38283 |  |  | *arborea* | 0.338 |
|  | nuclear hybrids | 1.00 | 1.90 | 1.4091 | 0.24671 |  |  | *arborea* | 1.000 |
|  | cyto-nuclear hybrids |  |  |  |  |  |  | *orientalis* | 0.854 |
|  | nuclear hybrids |  |  |  |  |  |  | cyto-nuclear hybrids | 0.514 |
| Do_Dn | *H. arborea* | 1.30 | 3.00 | 2.3295 | 0.37164 | 22.012 | < 0.001 | *orientalis* | 0.000 |
|  | *H. orientalis* | 2.10 | 3.90 | 2.8839 | 0.41331 |  |  | cyto-nuclear hybrids | 0.926 |
|  | cyto-nuclear hybrids | 2.10 | 3.70 | 2.7565 | 0.43257 |  |  | *arborea* | 0.000 |
|  | nuclear hybrids | 2.30 | 3.50 | 2.8227 | 0.35980 |  |  | *arborea* | 0.000 |
|  | cyto-nuclear hybrids |  |  |  |  |  |  | *orientalis* | 0.926 |
|  | nuclear hybrids |  |  |  |  |  |  | cyto-nuclear hybrids | 0.943 |
| Dop | *H. arborea* | 3.20 | 4.50 | 3.9770 | 0.32062 | 3.534 | 0.016 | *orientalis* | 0.052 |
|  | *H. orientalis* | 2.90 | 5.20 | 4.1696 | 0.45163 |  |  | cyto-nuclear hybrids | 0.837 |
|  | cyto-nuclear hybrids | 3.50 | 5.20 | 4.0826 | 0.43761 |  |  | *arborea* | 0.032 |
|  | nuclear hybrids | 3.40 | 4.90 | 4.2545 | 0.44156 |  |  | *arborea* | 0.708 |
|  | cyto-nuclear hybrids |  |  |  |  |  |  | *orientalis* | 0.837 |
|  | nuclear hybrids |  |  |  |  |  |  | cyto-nuclear hybrids | 0.483 |
| Dn | *H. arborea* | 2.00 | 3.20 | 2.7705 | 0.27285 | 17.413 | < 0.001 | *orientalis* | 0.000 |
|  | *H. orientalis* | 2.60 | 3.60 | 3.1250 | 0.26985 |  |  | cyto-nuclear hybrids | 0.169 |
|  | cyto-nuclear hybrids | 2.40 | 3.50 | 2.9696 | 0.24390 |  |  | *arborea* | 0.008 |
|  | nuclear hybrids | 2.60 | 3.50 | 2.9864 | 0.26421 |  |  | *arborea* | 0.014 |
|  | cyto-nuclear hybrids |  |  |  |  |  |  | *orientalis* | 0.169 |
|  | nuclear hybrids |  |  |  |  |  |  | cyto-nuclear hybrids | 0.997 |
| Lo | *H. arborea* | 3.30 | 6.20 | 4.1164 | 0.50437 | 3.607 | 0.015 | *orientalis* | 0.148 |
|  | *H. orientalis* | 3.40 | 5.10 | 4.2911 | 0.37575 |  |  | cyto-nuclear hybrids | 0.386 |
|  | cyto-nuclear hybrids | 3.80 | 5.70 | 4.4217 | 0.56081 |  |  | *arborea* | 1.000 |
|  | nuclear hybrids | 3.80 | 4.70 | 4.1136 | 0.22317 |  |  | *arborea* | 0.028 |
|  | cyto-nuclear hybrids |  |  |  |  |  |  | *orientalis* | 0.386 |
|  | nuclear hybrids |  |  |  |  |  |  | cyto-nuclear hybrids | 0.095 |

**TABLE S2a**

**Grouping (i): Variable data for a principal component analysis of body measurements of *Hyla arborea*. *H. orientalis* and their pooled hybrids**. Eigenvalues and percentages (%) of variation, explained by each of three axes; variables that load strongly (>0.7) on PC in **bold**. (1: *H. arborea,* N = 72; 2: *H. orientalis*, N = 66; 3: pooled hybrids, N = 53)

|  | **Principal components** | | |
| --- | --- | --- | --- |
|  | 1 | 2 | 3 |
| SVL | **0.892** | -0.064 | -0.193 |
| LC | **0.832** | 0.117 | -0.192 |
| LM | **0.773** | -0.257 | -0.092 |
| T | **0.902** | -0.209 | -0.173 |
| F | **0.832** | -0.287 | -0.153 |
| LP | **0.865** | -0.259 | -0.091 |
| LTym | 0.484 | **0.709** | -0.236 |
| Do_Dtym | 0.215 | **0.789** | -0.043 |
| Do_Dn | 0.454 | 0.048 | **0.721** |
| Dop | 0.502 | 0.182 | 0.483 |
| Dn | 0.560 | -0.260 | 0.421 |
| Lo | 0.617 | 0.533 | 0.134 |
| Eigenvalue | 5.772 | 1.791 | 1.151 |
| % | 48.1 | 14.9 | 9.6 |

**TABLE S2b**

**Grouping (ii): Variable data for a principal component analysis of body measurements of *Hyla arborea*. *H. orientalis* and their pooled hybrids (specimens with an SVL >39 mm)**. Eigenvalues and percentages (%) of variation, explained by each of three axes. Variables that load strongly (>0.7) on PC in **bold** (1: *H. arborea,* N = 61; 2: *H. orientalis*, N = 56; 3: pooled hybrids, N = 45).

**Grouping (iii): Variable data for a principal component analysis of body measurements of *Hyla arborea*. *H. orientalis* and two types of hybrids (“nuclear hybrids” and “cytonuclear hybrids”; specimens with an SVL >39 mm)**.**)** and Eigenvalues and percentage (%) of variation explained by each of three axes. Variables that load strongly (>0.7) on PC in bold. (1: *H. arborea,* N = 61; 2: *H. orientalis* N = 56; 3: nuclear hybrids, N = 23; 4: cytonuclear hybrids, N = 22)

|  | Principal components | | |
| --- | --- | --- | --- |
|  | 1 | 2 | 3 |
| SVL | **0.768** | 0.029 | -0.294 |
| LC | 0.659 | 0.291 | -0.237 |
| LM | 0.584 | -0.289 | -0.016 |
| T | **0.826** | -0.255 | -0.238 |
| F | **0.752** | -0.330 | -0.186 |
| LP | **0.845** | -0.298 | -0.046 |
| LTym | 0.280 | **0.787** | -0.215 |
| Do_Dtym | 0.194 | **0.744** | -0.078 |
| Do_Dn | 0.429 | **0.078** | 0.678 |
| Dop | 0.417 | 0.229 | 0.521 |
| Dn | 0.442 | -0.269 | 0.515 |
| Lo | 0.488 | 0.596 | 0.219 |
| Eigenvalue | 4.234 | 2.09 | 1.332 |
| % | 35.3 | 17.4 | 11.1 |

**TABLE S3a** Significance levels for the PCA on grouping (i): on *H. arborea*. *H. orientalis*

and their pooled types of hybrids (grouping i)

(1: *H. arborea,* N = 72; 2: *H. orientalis*, N = 66; 3: pooled hybrids, N = 53)

| MANOVA test. Wilks’ lambda = 0.57. F = 17.796. p < 0.001 | | |
| --- | --- | --- |
| **PC** | **Groups distinguishable** | ***p*** |
| PC1 | *H. arborea* *vs*. *H. orientalis* | 0.001 |
| PC2 | *H. arborea* *vs*. *H. orientalis* | < 0.001 |
|  | *H. orientalis* *vs*. hybrids | 0.045 |
| PC3 | *H. arborea* *vs*. *H. orientalis* | < 0.001 |
|  | *H. arborea* *vs*. hybrids | < 0.001 |

**TABLE S3b** Significance levels for the PCA on grouping (i): on *H. arborea*. *H. orientalis*

and their pooled types of hybrids (grouping ii).

(1: *H. arborea,* N = 61; 2: *H. orientalis*, N = 56; 3: pooled hybrids, N = 45)

| MANOVA test, Wilks’ lambda = 0.44, F = 26.546, p < 0.001 | | |
| --- | --- | --- |
| **PC** | **Groups distinguishable** | ***p*** |
| PC1 | *H. arborea* *vs*. *H. orientalis* | < 0.001 |
|  | *H. arborea* *vs*. hybrids | 0.007 |
|  | *H. orientalis* vs. hybrids | 0.019 |
| PC2 | *H. arborea* *vs*. *H. orientalis* | < 0.001 |
|  | *H. arborea vs.* hybrids | 0.007 |
| PC3 | *H. arborea* *vs*. *H. orientalis* | < 0.001 |
|  | *H. arborea* *vs*. hybrids | < 0.001 |

**TABLE S3c** Significance levels for the PCA on grouping (iii): on *H. arborea*. *H. orientalis*

and two types of hybrids (“nuclear hybrids” and “cytonuclear hybrids”) (grouping iii).

(1: *H. arborea,* N = 61; 2: *H. orientalis* N = 56; 3: nuclear hybrids, N = 23;

4: cytonuclear hybrids, N = 22)

| MANOVA test, Wilks’ lambda = 0.422, F = 17.976, p < 0.001 | | |
| --- | --- | --- |
| **PC** | **Groups distinguishable** | ***p*** |
| PC1 | *H. arborea* *vs*. *H. orientalis* | < 0.001 |
|  | *H. arborea* *vs*. nucl. hybrids | 0.025 |
| PC2 | *H. arborea* *vs*. *H. orientalis* | < 0.001 |
|  | *H. arborea vs.* cytonuclear hybrids | 0.003 |
| PC3 | *H. arborea* *vs*. *H. orientalis* | < 0.001 |
|  | *H. arborea* *vs*. nucl. hybrids | 0.002 |
|  | *H. arborea vs.* cytonuclear hybrids | < 0.001 |

**TABLE S4a** (grouping i): Summary of discriminant function analysis for *H. arborea*, *H. orientalis* and their pooled hybrids. (*H. arborea*, N = 72; *H. orientalis*, N = 66; hybrids, N = 53)

|  | **Wilks’ Lambda** | **F** | **df1** | **df2** | **p** |
| --- | --- | --- | --- | --- | --- |
| SVL | 0.988 | 1.120 | 2 | 188 | 0.329 |
| LC | 0.968 | 3.137 | 2 | 188 | 0.046 |
| LM | 0.968 | 3.139 | 2 | 188 | 0.046 |
| T | 0.913 | 8.931 | 2 | 188 | 0.000 |
| F | 0.907 | 9.612 | 2 | 188 | 0.000 |
| LP | 0.877 | 13.185 | 2 | 188 | 0.000 |
| LTym | 0.939 | 6.080 | 2 | 188 | 0.003 |
| Do_Dtym | 0.950 | 4.908 | 2 | 188 | 0.008 |
| Do_Dn | 0.723 | 36.059 | 2 | 188 | 0.000 |
| Dop | 0.961 | 3.822 | 2 | 188 | 0.024 |
| Dn | 0.844 | 17.425 | 2 | 188 | 0.000 |
| Lo | 0.978 | 2.091 | 2 | 188 | 0.126 |

**TABLE S4b** (grouping (ii): Summary of discriminant function analysis for *H. arborea*, *H. orientalis* and their pooled hybrids, only specimens with SVL >39 mm. (*H. arborea,* N = 61; *H. orientalis*, N = 56; hybrids N = 45)

|  | **Wilks’ lambda** | **F** | **df1** | **df2** | **p** |
| --- | --- | --- | --- | --- | --- |
| SVL | 0.972 | 2.262 | 2 | 159 | 0.107 |
| LC | 0.961 | 3.226 | 2 | 159 | 0.042 |
| LM | 0.932 | 5.760 | 2 | 159 | 0.004 |
| T | 0.801 | 19.785 | 2 | 159 | 0.000 |
| F | 0.834 | 15.847 | 2 | 159 | 0.000 |
| LP | 0.701 | 33.861 | 2 | 159 | 0.000 |
| LTym | 0.915 | 7.350 | 2 | 159 | 0.001 |
| Do_Dtym | 0.933 | 5.690 | 2 | 159 | 0.004 |
| Do_Dn | 0.707 | 33.001 | 2 | 159 | 0.000 |
| Dop | 0.949 | 4.251 | 2 | 159 | 0.016 |
| Dn | 0.752 | 26.255 | 2 | 159 | 0.000 |
| Lo | 0.968 | 2.616 | 2 | 159 | 0.076 |

**TABLE S4c** (grouping (iii): Summary of discriminant function analysis for *H. arborea*, *H. orientalis* and two types of hybrids (“nuclear hybrids” and “cytonuclear hybrids”), only specimens with SVL >39 mm.

(*H. arborea,* N = 61; *H. orientalis*, N = 56; nuclear hybrids, N = 23; cytonuclear hybrids, N = 22)

|  | **Wilks’ lambda** | **F** | **df1** | **df2** | **p** |
| --- | --- | --- | --- | --- | --- |
| SVL | 0.969 | 1.658 | 3 | 158 | 0.178 |
| LC | 0.952 | 2.628 | 3 | 158 | 0.052 |
| LM | 0.927 | 4.161 | 3 | 158 | 0.007 |
| T | 0.800 | 13.143 | 3 | 158 | 0.000 |
| F | 0.833 | 10.592 | 3 | 158 | 0.000 |
| LP | 0.701 | 22.516 | 3 | 158 | 0.000 |
| LTym | 0.903 | 5.653 | 3 | 158 | 0.001 |
| Do_Dtym | 0.922 | 4.449 | 3 | 158 | 0.005 |
| Do_Dn | 0.705 | 22.012 | 3 | 158 | 0.000 |
| Dop | 0.937 | 3.534 | 3 | 158 | 0.016 |
| Dn | 0.752 | 17.413 | 3 | 158 | 0.000 |
| Lo | 0.936 | 3.607 | 3 | 158 | 0.015 |

**TABLE S5a**

**Grouping (i): Re-classification matrix of a discriminant function analysis on grouping (i) *H. arborea*. *H. orientalis* and their pooled types of hybrids.** (*H. arborea* N = 72, *H. orientalis* N = 66, hybrids N = 53)

Two canonical vectors were extracted from the data. Axis 1 (Chi-square test: 179.621, *p* < 0.001) explained 91.3 % (Eigenvalue = 1.368) of the variation and the Axis 2 (Chi-square test: 22.274, *p* = 0.022) explained 8.7% (Eigenvalue = 0.13) of the variation (Figure S2a; Table S4a).

*H. orientalis* and hybrids do not differ, whereas all other are well separated along the Axis 1 (*H. arborea* vs. *H. orientalis*: p < 0.001, *H. arborea* vs. hybrids: *p* < 0.001, *H. orientalis* vs. hybrids: *p* = 0.067), whereas along the Axis 2 *H. arborea* and *H. orientalis* do not differ (*H. arborea* vs. *H. orientalis*: p = 0.188, *H. arborea* vs. hybrids: *p* < 0.001, *H. orientalis* vs. hybrids: *p* < 0.001).

|  | **Groups** | **Predicted group membership** | | | Total |
| --- | --- | --- | --- | --- | --- |
|  |  | *H. arborea* | *H. orientalis* | Hybrids |  |
| Numbers | *H. arborea* | 60 | 4 | 8 | 72 |
|  | *H. orientalis* | 4 | 47 | 15 | 66 |
|  | hybrids | 8 | 13 | 32 | 53 |
| % | *H. arborea* | 83.3 | 5.6 | 11.1 | 100.0 |
|  | *H. orientalis* | 6.1 | 71.2 | 22.7 | 100.0 |
|  | Hybrids | 15.1 | 24.5 | 60.4 | 100.0 |

**TABLE S5b**

**Grouping (ii): Re-classification matrix of a discriminant function analysis on grouping (i) *H. arborea*. *H. orientalis* and their pooled types of hybrids.** (*H. arborea* N = 61, *H. orientalis* N = 56, hybrids N = 45).

Two canonical vectors were extracted from the data. Axis 1 (Chi-square test: 188.959, p < 0.001) explained 92.3 % (Eigenvalue = 1.946) of the variation and the Axis 2 (Chi-square test: 23.119, p = 0.017) explained 7.7% (Eigenvalue = 0.124) of the variation (Figure S2b, Table S4b).

The following forms are separated along the Axis 1 (*H. arborea* vs. *H. orientalis*: p < 0.001, *H. arborea* vs. hybrids: p < 0.001, *H. orientalis* vs. hybrids: p = 0.003), whereas along the Axis 2 *H. arborea* and *H. orientalis* do not differ (*H. arborea* vs*. H. orientalis* p = 0.267, *H. arborea* vs. hybrids p = 0.001, *H. orientalis* vs. hybrids: p < 0.001).

|  | **Groups** | **Predicted group membership** | | | Total |
| --- | --- | --- | --- | --- | --- |
|  |  | *H. arborea* | *H. orientalis* | hybrids |  |
| Numbers | *H. arborea* | 52 | 3 | 6 | 61 |
|  | *H. orientalis* | 3 | 41 | 12 | 56 |
|  | Hybrids | 3 | 11 | 31 | 45 |
| % | *H. arborea* | 85.2 | 4.9 | 9.8 | 100.0 |
|  | *H. orientalis* | 5.4 | 73.2 | 21.4 | 100.0 |
|  | hybrids | 6.7 | 24.4 | 68.9 | 100.0 |

**TABLE S5c**

**Grouping (iii): Re-classification matrix of a discriminant function analysis on grouping (i) *H. arborea*. *H. orientalis* and two types of hybrids (“nuclear hybrids” and “cytonuclear hybrids”).** (*H. arborea* N = 61, *H. orientalis* N = 56, nuclear hybrids N = 23, cytonuclear hybrids N = 22). Three canonical vectors were extracted from the data. Axis 1 (Chi-square test: 201.141, p < 0.001) explained 89.1 % (Eigenvalue = 1.969) of the variation, the Axis 2 (Chi-square test 34.631, p = 0.042) explained 7.4 % (Eigenvalue = 0.163) of the variation, and the Axis 3 (Chi-square test: 11.584, p = 0.314) explained 3.6 % (Eigenvalue = 0.079) of the variation (Fig. S2c, Table 4c). The following forms are distinguishable along the Axis 1: *H. arborea* vs. *H. orientalis*: p < 0.001, *H. arborea* vs. nuclear hybrids: p < 0.001, *H. orientalis* vs. cyto-nuclear hybrids: p < 0.001, *H. orientalis* vs. nuclear hybrids: p = 0.005; along the Axis 2: *H. arborea* vs. nuclear hybrids: p = 0.026, *H. arborea* vs. cyto-nuclear hybrids: p = 0.022, *H. orientalis* vs. nuclear hybrids: p = 0.001, *H. orientalis* vs cyno-nuclear hybrids p = 0.001; along the Axis 3: Nuclear hybrids vs. cyto-nuclear hybrids: p = 0.003.

|  | **Groups** | **Predicted group membership** | | | | Total |
| --- | --- | --- | --- | --- | --- | --- |
|  |  | *H. arborea* | *H. orientalis* | Nuclear hybrids | Cyto-nuclear hybrids |  |
| Numbers | *H. arborea* | 51 | 20 | 6 | 2 | 61 |
|  | *H. orientalis* | 2 | 37 | 6 | 11 | 56 |
|  | Nuclear hybrids | 2 | 4 | 12 | 5 | 23 |
|  | Cyto-nuclear  hybrids | 1 | 4 | 3 | 14 | 22 |
| % | *H. arborea* | 83.6 | 3.3 | 9.8 | 3.3 | 100.0 |
|  | *H. orientalis* | 3.6 | 66.1 | 10.7 | 19.6 | 100.0 |
|  | Nuclear hybrids | 8.7 | 17.4 | 52.2 | 21.7 | 100.0 |
|  | Cyto-nuclear  hybrids | 4.5 | 18.2 | 13.6 | 63.6 | 100.0 |

**TABLE S6a** **Grouping (i): Re-classification matrix of a discriminant function analysis on grouping (i) *H. arborea*. *H. orientalis* and their pooled types of hybrids.**

Discriminant function (Eigenvalue 1.825, Wilks’ Lambda 0.354, Chi-square = 135.013, p < 0.001)

|  | **Groups** | **Predicted group membership** | | Total |
| --- | --- | --- | --- | --- |
|  |  | *H. arborea* | *H. orientalis* |  |
| Numbers | *H. arborea* | 66 | 6 | 72 |
|  | *H. orientalis* | 9 | 57 | 66 |
|  | Hybrids | 10 | 43 | 53 |
| % | *H. arborea* | 91.7 | 8.3 | 100.0 |
|  | *H. orientalis* | 13.6 | 86.4 | 100.0 |
|  | Hybrids | 18.9 | 81.1 | 100.0 |

**TABLE S6b** **Grouping (ii): Re-classification matrix of a discriminant function analysis on grouping (i) *H. arborea*. *H. orientalis* and their pooled types of hybrids.** (> 39 mm).

Discriminant function (Eigenvalue 2.396, Wilks’ Lambda 0.294, Chi-square = 133.265, p < 0.001)

|  | **Groups** | **Predicted group membership** | | Total |
| --- | --- | --- | --- | --- |
|  |  | *H. arborea* | *H. orientalis* |  |
| Numbers | *H. arborea* | 56 | 5 | 61 |
|  | *H. orientalis* | 3 | 53 | 56 |
|  | Hybrids | 8 | 37 | 45 |
| % | *H. arborea* | 91.8 | 8.2 | 100.0 |
|  | *H. orientalis* | 5.4 | 94.6 | 100.0 |
|  | Hybrids | 17.8 | 82.2 | 100.0 |

**TABLE S6c** **Grouping (iii): Re-classification matrix of a discriminant function analysis on grouping (i) *H. arborea*. *H. orientalis* and two types of hybrids (“nuclear hybrids” and “cytonuclear hybrids”).** (>39mm). 1 discriminant function (eigenvalue 2.396, Wilks’ Lambda 0.294, Chi-square = 133.265, p<0.001)

|  | **Groups** | **Predicted group membership** | | Total |
| --- | --- | --- | --- | --- |
|  |  | *H. arborea* | *H. orientalis* |  |
| Numbers | *H. arborea* | 56 | 5 | 61 |
|  | *H. orientalis* | 3 | 53 | 56 |
|  | Nuclear hybrids | 6 | 17 | 23 |
|  | cyto-nuclear hybrids | 2 | 20 | 22 |
| % | *H. arborea* | 91.8 | 8.2 | 100.0 |
|  | *H. orientalis* | 5.4 | 94.6 | 100.0 |
|  | Nuclear hybrids | 26.1 | 73.9 | 100.0 |
|  | cyto-nuclear hybrids | 9.1 | 90.9 | 100.0 |
